# An mHealth Pain Coping Skills Training Intervention for Hematopoietic Stem Cell Transplantation Patients: Development and Pilot Randomized Controlled Trial

**DOI:** 10.2196/mhealth.8565

**Published:** 2018-03-19

**Authors:** Tamara J Somers, Sarah A Kelleher, Caroline S Dorfman, Rebecca A Shelby, Hannah M Fisher, Krista Rowe Nichols, Keith M Sullivan, Nelson J Chao, Gregory P Samsa, Amy P Abernethy, Francis J Keefe

**Affiliations:** ^1^ Department of Psychiatry and Behavioral Sciences Duke University Medical Center Durham, NC United States; ^2^ Division of Hematologic Malignancies and Cellular Therapy Duke University Medical Center Durham, NC United States; ^3^ Department of Biostatistics and Bioinformatics Duke University Medical Center Durham, NC United States; ^4^ Duke Cancer Institute Duke University Medical Center Durham, NC United States

**Keywords:** stem cell transplantation, hematopoietic stem cell, cancer-related pain, coping skills

## Abstract

**Background:**

Pain is a challenge for patients following hematopoietic stem cell transplantation (HCT).

**Objective:**

This study aimed to develop and test the feasibility, acceptability, and initial efficacy of a Web-based mobile pain coping skills training (mPCST) protocol designed to address the needs of HCT patients.

**Methods:**

Participants had undergone HCT and reported pain following transplant (N=68). To guide intervention development, qualitative data were collected from focus group participants (n=25) and participants who completed user testing (n=7). After their input was integrated into the mPCST intervention, a pilot randomized controlled trial (RCT, n=36) was conducted to examine the feasibility, acceptability, and initial efficacy of the intervention. Measures of acceptability, pain severity, pain disability, pain self-efficacy, fatigue, and physical disability (self-report and 2-min walk test [2MWT]) were collected.

**Results:**

Participants in the focus groups and user testing provided qualitative data that were used to iteratively refine the mPCST protocol. Focus group qualitative data included participants’ experiences with pain following transplant, perspectives on ways to cope with pain, and suggestions for pain management for other HCT patients. User testing participants provided feedback on the HCT protocol and information on the use of videoconferencing. The final version of the mPCST intervention was designed to bridge the intensive outpatient (1 in-person session) and home settings (5 videoconferencing sessions). A key component of the intervention was a website that provided personalized messages based on daily assessments of pain and activity. The website also provided intervention materials (ie, electronic handouts, short videos, and audio files). The intervention content included pain coping advice from other transplant patients and instructions on how to apply pain coping skills while engaging in meaningful and leisure activities. In the RCT phase of this research, HCT patients (n=36) were randomized to receive the mPCST intervention or to proceed with the treatment as usual. Results revealed that the mPCST participants completed an average of 5 out of 6 sessions. The participants reported that the intervention was highly acceptable (mean 3/4), and they found the sessions to be helpful (mean 8/10) and easy to understand (mean 7/7). The mPCST participants demonstrated significant improvements in pre- to post-treatment pain, self-efficacy (*P*=.03, *d*=0.61), and on the 2MWT (*P*=.03, *d*=0.66), whereas the patients in the treatment-as-usual group did not report any such improvements. Significant changes in pain disability and fatigue were found in both groups (multiple *P*<.02); the magnitudes of the effect sizes were larger for the mPCST group than for the control group (pain disability: *d*=0.79 vs 0.69; fatigue: *d*=0.94 vs 0.81). There were no significant changes in pain severity in either group.

**Conclusions:**

Using focus groups and user testing, we developed an mPCST protocol that was feasible, acceptable, and beneficial for HCT patients with pain.

**Trial Registration:**

ClinicalTrials.gov NCT01984671; https://clinicaltrials.gov/ct2/show/NCT01984671 (Archived by WebCite at http://www.webcitation.org/6xbpx3clZ)

## Introduction

### Background

Persistent pain is a major challenge for patients following hematopoietic stem cell transplantation (HCT) [1-5]. This pain can be related to diverse sources including patients’ disease, treatment regimens, medications, or pre-existing conditions [1]. HCT patients may experience multiple sources of pain including joint, bone, headache, mouth, gastro-intestinal, neuropathic, and thoracic pain [1,6]. Moderate to severe pain has been reported by 30-58% of HCT patients [4,5,7,8]. Pain occurs before, acutely following, and in the months and years following the transplant [4-6]. Persistent pain in patients with HCT is related to lower levels of physical functioning, less energy, and more psychosocial problems [4,7,9,10]. There are limitations of analgesic regimens in HCT patients (eg, side effects and limited relief); there is a clear need for adjuvant strategies to manage pain in these patients [11].

### Psychosocial Pain Interventions

Psychosocial interventions that teach patients with chronic diseases skills to manage their pain can improve their abilities to cope with and reduce the pain [9,10]. Addressing psychosocial, cognitive, and behavioral factors related to persistent pain may be especially important for HCT patients [12-14] who face unique challenges and may have particularly low levels of confidence in their abilities to control their pain (ie, self-efficacy for pain control) [15,16]. HCT patients with low self-efficacy for pain control are more likely to report high levels of pain disability and other bothersome symptoms [17,18]. As such, a psychosocial pain intervention designed to help HCT patients manage their pain in the context of their unique pain-related challenges may prove to be particularly beneficial.

Psychosocial pain protocols are typically delivered in-person, require patients to travel to a medical center setting, and/or are not tailored to the unique challenges faced by HCT patients [19-21]. Patients undergoing HCT face substantial burden resulting from the life-threatening, chronic illness that has led to HCT, invasive treatment regimens (including HCT), and interruptions to their normal routines and functioning. When undergoing HCT, patients are required to spend several days pre- and post-transplant in an inpatient or intensive outpatient setting. Then, patients and their caregivers transition to temporary housing that is in close proximity to the medical center to receive several weeks of daily outpatient care. Finally, after weeks of intense care in the medical center setting, they are discharged home as they continue to recover, often many miles from the clinic. These unique challenges can increase pain and make pain management particularly difficult. Using mobile health (mHealth) technologies to deliver psychosocial pain interventions may increase the feasibility, acceptability, and efficacy of these interventions for HCT patients.

### Study Objectives

The objective of this line of research was to develop a mobile health pain coping skills training (mPCST) protocol, designed to address the specific pain and psychosocial challenges of HCT patients. We used an iterative development model to design the protocol; methods from grounded theory were followed [22]. First, we developed an initial mPCST intervention for HCT patients using our study team’s expertise and experience in PCST, cognitive-behavioral pain interventions, mobile health technology, and the treatment of HCT. We then conducted focus groups with both HCT patients and providers to refine and adapt the intervention. Following this, the enhanced intervention was delivered to a separate small group of patients who completed user testing. Qualitative data gathered from each stage of development were used to inform the subsequent modification of the intervention.

A pilot randomized controlled trial (RCT) was conducted to examine the feasibility, acceptability, and initial efficacy of the final mPCST protocol. The first aim of the pilot RCT was to show that the mPCST protocol would be feasible (ie, accrual, attrition, and adherence) and acceptable. The second aim was to examine the initial efficacy of the mPCST protocol (compared with a treatment as usual condition) on pain severity, pain disability, pain self-efficacy, fatigue, and physical disability (ie, self-report and 2-min walk test [2MWT]).

## Methods

### Participants

All participants were recruited from the adult bone marrow transplant clinic (ABMT) at a major academic medical center. Eligible patients were >21 years old, had cancer that led to transplant, and had at least one clinical post-transplant pain score of ≥3/10. Exclusion criteria included cognitive impairment (eg, dementia, psychosis) and inability to converse in English. Eligible health care providers were recruited through ABMT administrators and included nurse practitioners, physician’s assistants, and registered nurses.

### Development of the Mobile Pain Coping Skills Training Protocol

An initial mPCST protocol was developed that was informed by the investigators’ expertise in several areas including PCST protocol development [9,23-31], mHealth applications [32-34], and observational studies of pain and other symptoms in HCT patients [8,35]. Traditional PCST protocols have been delivered over several weekly sessions (eg, 6-12), conducted face-to-face at a major medical center or sometimes by telephone, and are often about an hour long [36-39].

Most PCST protocols include some combination of the following content and skills training. A rationale is provided [40,41] to help patients understand that pain is a complex experience influenced by thoughts, feelings, and behaviors. Patients are taught several skills (eg, relaxation training, cognitive-restructuring, activity pacing, pleasant activity planning, imagery, problem solving, and goal setting) [21,23,31,42] to enhance their ability to cope with their pain by changing their thoughts, feelings, and behaviors. Sessions focus on reviewing content and skills practice from the previous session, learning a new skill, skill rehearsal, and home skills practice planning for the upcoming week.

The initial mPCST protocol we proposed included 6, 50-min sessions delivered over a period of 6-10 weeks. This protocol included several innovative features designed to address the unique challenges faced by HCT patients: it was brief, bridged the intensive outpatient (1 session) and home (5 sessions) settings, and used videoconferencing via an iPad for delivery in patients’ homes. The initial mPCST session was designed to occur in-person in the medical center setting before discharge home to allow facilitation of a relationship between the therapist and patient, and to establish care that bridges intensive outpatient and home care. The subsequent 5 sessions were to be delivered to the patients in their home environment through the use of videoconferencing (iPad and Skype). Videoconferencing (vs in-person and/or telephone delivery) provided the following important advantages: (1) it allowed patients who lived far from the medical center to engage in the intervention, (2) there is evidence that educational and psychosocial content is better communicated through videoconferencing than through telephone [43], and (3) social cognitive theory suggests videoconferencing could lead to improvements in pain self-efficacy by having patients practice and receive feedback on skills in their home environment (ie, where they need to implement skills daily) [44].

The initial intervention included didactic and experiential components, which were summarized in handouts and on the study website. Homework on how to incorporate material from the session into daily life was also given to facilitate skill acquisition and generalization of skills use into their normal routine [45,46]. Adherence was promoted by reviewing homework at the beginning of each session, developing action plans for skills use, and providing positive reinforcement. The study website provided patients with a place to have a daily connection with the mPCST intervention by recording their symptoms and skills use, accessing study materials, and receiving tailored feedback based on their daily assessment of skills practice.

**Figure 1:**
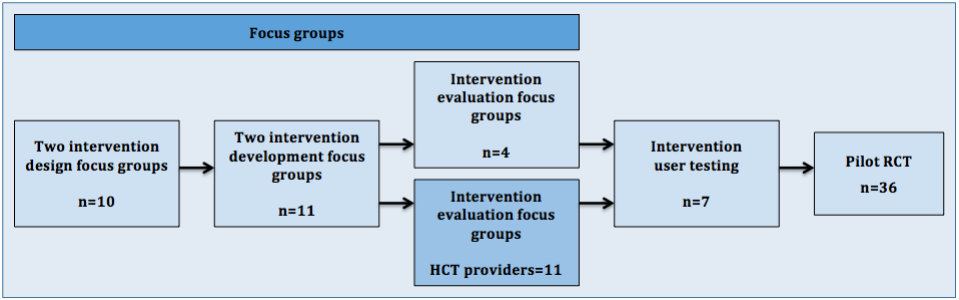
Mobile pain coping skills training (mPCST) for hematopoietic stem cell transplantation (HCT) focus groups, user testing, and randomized controlled trial (RCT) development process.

Figure 1 displays mPCST for the HCT focus groups, user testing, and RCT development process.

### Focus Groups

#### Intervention Design Focus Groups

Two participatory design focus groups (n=10) were conducted to guide investigators in tailoring the mPCST protocol to meet the unique needs of HCT patients with pain. Focus group guides were developed based on the investigators’ experience, past work, and the larger empirical literature. Group discussions focused on the patient’s experiences and challenges of HCT pain, intervention content, and intervention characteristics (eg, material types, topics).

#### Intervention Development Focus Groups

Two intervention development focus groups allowed participants (n=11) to evaluate the tailored mPCST protocol materials developed in the intervention design focus groups. These groups were conducted using both in-person examples and a visual demonstration of the iPad and Skype system; patients were asked to provide the research team with objective information on challenges in using and preferences for delivery using this modality.

#### Intervention Evaluation Focus Groups

The final two focus groups comprised HCT patients (n=4) and providers (n=10). Providers recruited for the focus groups were clinical providers in the ABMT clinic and had no other role in this study. Using the feedback from the intervention design and development focus groups, the evaluation focus group was geared toward the final refinement of the mPCST protocol and technology. Participants were provided with a description of the intervention protocol, were asked detailed questions about specific components of the protocol (eg, daily measures, iPad technology, and use of pedometer), and were provided with an example of the materials that would be given to the potential participants (eg, iPad, patient manual, and handouts). Providers were asked to review and provide feedback on the patient manual and session handouts, and to use the study website on the iPad.

### User Testing

User testing of the developed mPCST protocol was conducted with 7 participants who reported HCT-related pain. Participants completed the 6-session mPCST protocol and were asked to provide feedback following each session. The information collected was used to further refine the intervention protocol.

### Pilot Randomized Controlled Trial

A separate group of participants (n=36) were randomized to either the mPCST or treatment-as-usual, control group. Pre- (before randomization) and postintervention assessments included measuring pain severity, pain disability, pain self-efficacy, fatigue, and physical disability (ie, self-report, 2MWT). Self-report assessments were completed via the study website; the 2MWT was conducted at the medical center.

Participants randomized to mPCST completed the first session following their baseline study assessment and before discharge home. The time between the baseline assessment and first session was on average 4.5 days (SD 9) and from the first session in the hospital to the first session at home was on average 10.5 days (SD 10). Participants who did not have Internet access to participate in videoconferencing and Web-based assessments from home or who desired to have study-provided hardware were loaned a tablet computer (ie, iPad) equipped with 3G Internet access; most participants elected to use the study-provided iPad to complete the intervention. Participants randomized to treatment as usual were also loaned a tablet computer as needed to complete assessments.

### Measures

Pilot RCT participants completed self-reported measures of acceptability, pain severity, pain disability, pain self-efficacy, fatigue, and physical disability. Demographic and medical variables were also collected.

#### Feasibility

Feasibility was assessed by examining overall accrual, attrition, and adherence.

#### Acceptability

Acceptability was assessed with the client satisfaction questionnaire (CSQ), 10-item version [47]. CSQ was completed by participants in the mPCST intervention group at the post-treatment assessment. The measure was shown to have good reliability (Cronbach alpha=.96).

#### Pain Severity

Pain severity was assessed with the 4-item brief pain inventory [48]. Patients rated their pain from 0=no pain to 10=worst pain imaginable in response to average pain, worst pain, least pain, and pain right now over the past 7 days. An average of the 4 items was used to create a single pain severity score. Internal consistency at pretreatment was found to be Cronbach alpha=.90.

#### Pain Disability

Pain disability was measured with the pain disability index [49]. This 7-item scale measures the degree of a patient’s disability within 7 life domains and has demonstrated good reliability and validity [49]. Internal consistency at pretreatment was Cronbach alpha=.91.

#### Pain Self-Efficacy

Self-efficacy for pain control was measured using the 5-item self-efficacy for pain subscale of the chronic pain self-efficacy scale measure (pretreatment Cronbach alpha=.86) [50].

#### Fatigue

Fatigue was measured with the patient-reported outcomes measurement information system adult fatigue profile short form (6 items) [51,52]. This scale has good precision across different levels of fatigue and has demonstrated good reliability (>.90) [52]. Internal consistency in this sample at pretreatment was Cronbach alpha=.95.

#### Physical Disability

Self-reported physical disability was measured with the functional assessment of cancer therapy well-being scale (FACT; 7-items). FACT has demonstrated good internal consistency, criterion validity, and sensitivity to change [53]. Internal consistency at pretreatment in this sample was Cronbach alpha=.84. Physical disability was also assessed using an objective measure, the 2MWT. The 2MWT is a laboratory-based assessment of patients’ physical disability that measures the functional capacity for physical lifestyle activity. The 2MWT provides a self-paced, timed test of the total distance in meters that a patient is able to walk over a 2-min period, and it has been shown to be sensitive to change following medical treatments. The 2MWT has shown moderate correlations with physical disability [54].

### Analytic Strategy

Focus groups were audio-recorded and 2 group leaders took field notes. Audio files were transcribed by a member of the research study team; a second team member performed a quality check by replaying the audio file and editing the transcript as needed. Grounded theory methods were used to evaluate the data gathered from the focus groups. Audio recordings were reviewed using open coding (ie, in vivo) and memoing by 3 members of the study team to generate repeated concepts. These results were categorized into 5 major themes through selective coding methods.

For the RCT, descriptive statistics were calculated for demographic, medical, feasibility, study self-report variables (ie, acceptability, pain severity, pain disability, pain self-efficacy, fatigue, and physical disability), and 2MWT. Analysis of variance (ANOVA), chi-square, or Fisher exact tests were used, as appropriate, to examine baseline differences between groups on medical and demographic variables. Outcome analyses were conducted using both an intent-to-treat approach (last value carried forward) and complete case analysis. The results of both the analytic strategies were comparable. Results using the complete case analysis approach are presented below. Paired *t* tests were used to examine within group differences from baseline to follow-up on study outcome variables (ie, pain severity, pain disability, self-efficacy for pain management, fatigue, and physical disability). The magnitudes of the effect sizes were defined according to standard convention for Cohen *d* (small=0.2, medium=0.5, large=0.8) [55].

## Results

### Focus Groups and User Testing

Participants included 32 individuals with HCT pain who had undergone autologous (87%, 28/32) or allogeneic (13%, 4/32) stem cell transplant and reported having post-transplant pain. Participants were 50% female (16/32), 72% Caucasian (23/32), and were aged between 43 and 76 years (mean 61). Majority of the participants were married (84%, 27/32) and 53% (17/32) had a college degree or higher. Participants were on average 20 (SD 14) months post-transplant. The provider intervention evaluation focus group consisted of 10 providers, all of whom were females and held a position within nursing care for HCT patients (5 nurse practitioners, 2 clinical nurse specialists, 1 registered nurse, 1 outpatient clinic nurse manager, and 1 clinical research nurse).

### Focus Group Results

Review of focus group recordings and field notes revealed 5 overall themes. Each theme is presented and described below; theme order is reflective of the order of content presented to the focus group and not related to rank or importance. A description of how the intervention protocol was modified based on each theme is provided. Textbox 1 provides representative patient quotes related to each theme.

#### Theme 1: Pain Experiences Pre- and Post-Transplant and Strategies Used to Cope With Pain

Participants reported experiencing post-transplant pain. The majority also reported having pain before transplant, with some reporting increased pain following the transplant. Patients reported that reasons for pain were neuropathy, graft versus host disease symptoms, joint inflammation, and pre-existing osteoarthritis. Neuropathy was frequently reported in hands and feet, whereas osteoarthritis pain was most commonly described in the hips, knees, hands, and feet. Participants in focus groups reported that pain was worse when they were sedentary and improved when they were busy and moving. Notably, many participants also endorsed significant sleep or fatigue problems following transplant.

Participants reported using the following measures to manage their pain: taking pain medications, wearing supportive shoes, receiving gentle massages, participating in regular exercise (eg, physical therapy, chair yoga, stationary biking, walking, and elliptical training), relying on their faith (eg, attending church, prayer, and scripture reading), being active in volunteer work (eg, cancer groups), and participating in hobbies (eg, cooking or baking, gardening, reading, and playing a musical instrument). Participants acknowledged that participating in these activities helped to distract them from their pain. On the basis of this theme, information was added to the protocol that describes the pain experiences of other transplant patients and suggestions from other patients about helpful pain coping strategies.

#### Theme 2: Post-Transplant Activities and Limitations in Activities

**Figure 2:**
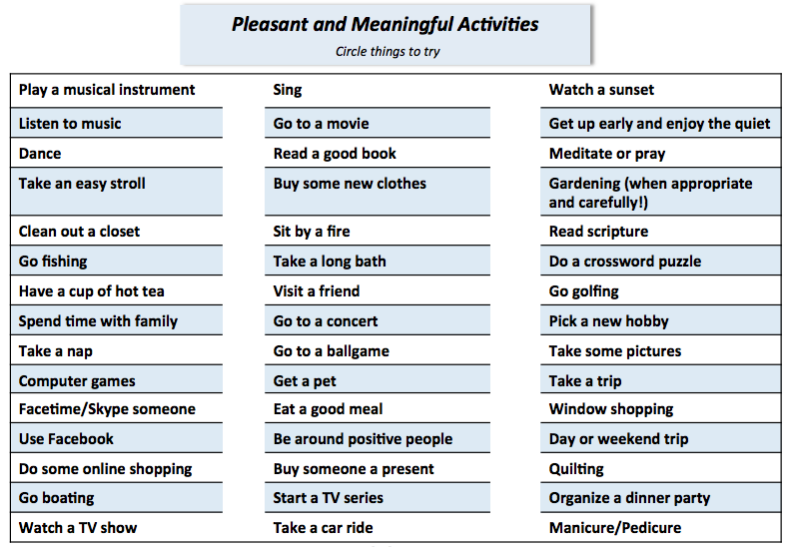
Intervention handout for pleasant and meaningful activities for hematopoietic stem cell transplantation (HCT) patients.

Participants in the intervention development focus groups were asked to brainstorm pleasant activities that they could begin again or start anew post-transplant. Participants listed playing a musical instrument, watching grandchildren, visiting friends and children, traveling, gardening, getting a pet, planning dinner parties, baking and cooking, getting manicures or pedicures, being more involved in their church, and volunteering. Interestingly, many participants also mentioned an increased post-transplant desire to participate in activities they find meaningful. Figure 2 displays patient intervention materials showing how this information was integrated into the study protocol.

Participants also stated that their pain limited their abilities to engage in leisure or recreational activities. In particular, several participants noted that their abilities to engage in daily exercises (eg, biking, running) had decreased or diminished. Participants also reported overdoing activities related to their work, family, and leisure time. Focus group participants recommended extending and expanding the information provided to participants on pleasant, meaningful, and leisure or recreational activities. This information was added to both the therapist protocol and patient handouts.

**Representative quotes from focus group participants related to each theme.**

Theme 1: Pain experiences and pain coping strategies

- It’s a hard balance [getting off pain meds] because you can’t just stop. When you scale back, we’re trying to work it out, so I’m gradually dosing down.
- Always wind up with my hips hurting the most [post-transplant].
- Honestly I can say I went from horrendous pain and can say I’m in no pain sitting here. It felt like a huge rubber band around my chest. And it was just as tight as it could possibly be. I couldn’t lie down; I would sit up and rock, just rock and pray, until I finally fell asleep.
- I wonder if my chemo and the whole situation didn’t just age my whole body, and the pains that I feel now might have been pains I wouldn’t feel, 5, 6, 17 years from now but that I think I’m feeling now.
- I have a lot of bone pain, but I had it before the transplant because the chemo was eating away at the bones in my back, so I’ve had some surgery on my back to help with that. The pain in my back got better during the transplant because I was in so much pain everywhere else.
- I’ve had some trouble with neuropathy. I still feel it, but I’m not taking anything for it.
- I’m feeling more pain now post-transplant. After the transplant was when I really started having the pain.
- Foot and leg cream—it has done wonders.
- Walking is the best thing for me when I have pain.
- I find that at night, even putting socks on makes a difference [in regards to the pain].
- I need to sit properly; if I didn’t have support in my lower back the pain is immediate. Breathing helps, in through the nose out through the mouth.

Theme 2: Activities and limitations in activities

- I was a very athletic person, and still am, I try to keep up with my kids. I had my walking stick, I had to stop and rest for a bit.
- I take breaks. I love baking Christmas cookies, but I can’t stand that long anymore. I’ll do a little bit; then take a break. Funny ways to do it and still get something accomplished.
- My husband bought me a piano for Christmas because I’ve always wanted to play it. I just forget everything. It is really a good relaxer.
- I love to garden, even on my worst days, I take a bucket and sit out there. Not only did I get something accomplished, but I did something I really enjoy.
- You’re finally home and you’re like I can do something, but you’re not allowed to.
- Right now I’m just getting out of seclusion so I’m not able to do much, but we’re planning to take trips.
- Now I have a totally different perspective on life, and it’s nice to get out and do something.

Theme 3: Pain-related cognitions

- I just try to deal with it.
- Sometimes I just live with it because I don’t want to take the pain meds.
- I feel better when I do anything.
- I made it through the first time, if I have to go it through again, I know what to expect and I can do it again.
- My cancer is back [in reference to thinking about the pain].
- How long is this [pain] going to last?
- It’s been 2 years, and if something strange happens [related to pain], I still worry.
- When you don’t really have any symptoms, it’s scary.

Theme 4: Advice for other transplant patients

- Don’t try to fight it [the pain]. Let the doctors know. It’s not their first rodeo.
- We’re not supposed to be proud at a time like this; we’re supposed to be honest.
- Would have been helpful to have spoken to some who had been through transplant beforehand.
- It does make you have to learn how to do things, such as learning how to take medication. And washing my hands, I’m good at that now.

Theme 5: Feedback on the mobile pain coping skills training (mPCST) protocol

- I’d call my daughter for help [with the iPad]. [I] wouldn’t try if she wasn’t there, she is a real techie. It could be managed with her.
- I can barely turn a computer on.
- I’ve been wearing a pedometer for about 3 years, so I wouldn’t have a problem. I do it already.
- I’d be willing to give this [the iPad and videoconferencing] a try with help.

#### Theme 3: Pain-Related Cognitions

Focus group participants were asked about their thoughts (negative and positive) surrounding their pain experience pre- and post-transplant. Many patients worried that their pain might indicate disease recurrence or progression. For example, participants endorsed the following cognitions: “What are they going to find at my next check-up appointment?,” “My cancer is back,” and “Something is wrong.” For others, thoughts were associated with perceptions of their pain and their abilities to reduce their pain. Negative thoughts included the following: “I want to get rid of this,” “How long will this pain last?,” “Will this ever go away?,” and “Why me?.” Overall, participants said that staying positive yet realistic was the best way for them to cope with these negative cognitions related to post-transplant pain. Positive thoughts that helped patients cope with pain and combat the aforementioned negative cognitions included the following: “This too shall pass,” “I am blessed,” and “This is my life right now.” Common negative and positive cognitions about pain provided by focus group participants were used as examples in the protocol during sessions with the therapist and in patient handouts.

#### Theme 4: Advice for Other Transplant Patients

**Figure 3:**
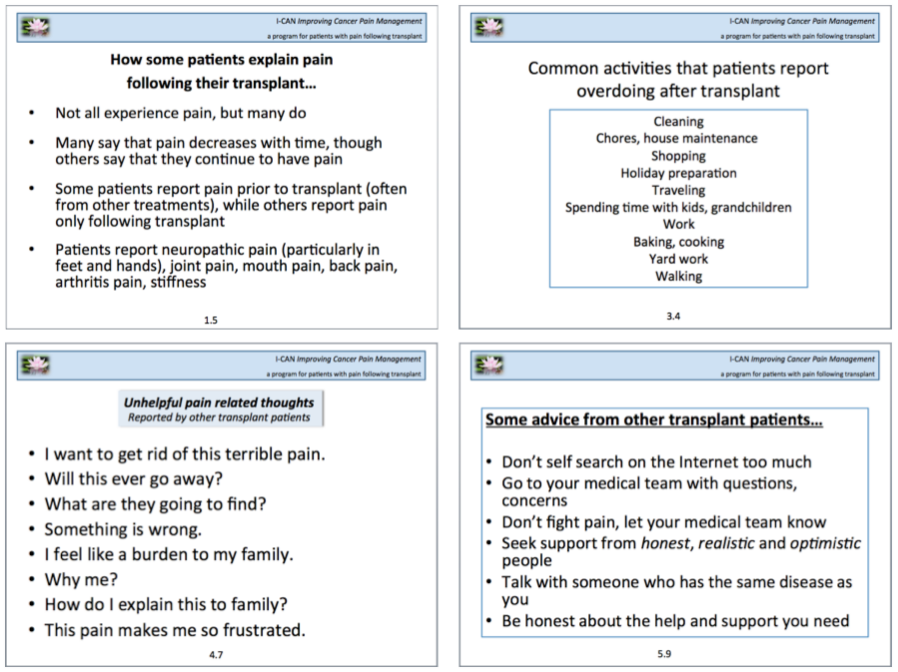
Intervention handout examples using advice from other transplant patients.

We asked participants what pain-related information they wish they had known before their own transplant. Group members acknowledged that it would have been helpful to receive more information on neuropathy and the different types of pain that might be experienced post-transplant. More broadly, participants expressed a desire for communication with prior transplant patients regarding the pain experience and general transplant-related information. Most of the participants agreed that having someone who had gone through a similar situation to talk to about their pain was helpful and comforting, and that such an outlet should be made available to HCT patients approaching transplant. On the basis of this consistent observation acknowledged across the focus groups, patient materials were updated to reflect advice from other transplant patients. The most common suggestion among HCT patients was to communicate any pain, discomfort, or other concerns to the medical team and caregivers rather than holding back. Group members advised future patients to be honest about their pain and about what physical and emotional help they needed. Figure 3 shows how the theme of advice for other transplant patients was incorporated into the patient handouts. Throughout the protocol, we used language that indicated that the information had come directly from other HCT patients.

#### Theme 5: Feedback on the Mobile Pain Coping Skills Training Protocol

Focus group participants provided feedback on the mPCST protocol and technology. The majority of participants agreed that they would be willing to use the iPad, Skype, and website technology with adequate instruction. Many of the participants reported that it would be helpful to have a connection with their medical team and a therapist during the transition to post-transplant life, as they often missed the daily contact with their medical team once discharged from daily care. This feedback reinforced our position that the most appropriate timing of this protocol was once the patient returned home. Participants’ responses also led to the inclusion of detailed instruction in the use of the technology (eg, iPad, Skype) for this study in the patient handouts.

### Provider Results

**Figure 4:**
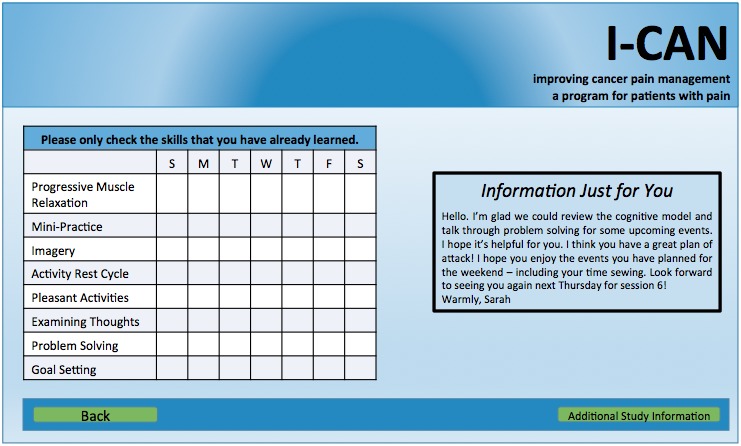
Intervention website page providing patient with tailored feedback.

Providers recommended that the study team collect daily fatigue levels in addition to daily pain scores and daily steps due to their strong observed relationship between pain and fatigue. Providers also expressed that it would be worthwhile to have participants wear a pedometer to track activity. One aspect of the website that was particularly popular with the providers was the *Just for You* feedback box designated for individualized therapist feedback for participants (See Figure 4). Providers also made suggestions about the appearance of the website (ie, use an easy to read font and color scheme).

Providers were asked how they currently help patients who experience post-transplant pain. The most common methods recommended by providers to deal with pain were medication and/or distraction. When providers recommended exercise, they frequently advised patients to use a recumbent bike, exercise bands for muscle strengthening, and yoga or chair yoga. Providers were asked to outline the most commonly reported challenges that patients face 5 to 10 weeks post-transplant besides pain. Fatigue, nutrition, and depression were listed as frequently encountered problems, and it was recommended that information be included in our protocol to help patients deal with such issues. For fatigue, providers underscored that patients should avoid going back to bed if tired; rather, they should take brief cat naps throughout the day. They also recommended establishing a routine to keep busy and making a point to get dressed every morning as well as pacing and prioritizing activities. Finally, when patients have pain, psychological issues such as depression are prevalent post-transplant, and providers recommended that study therapists be aware of this and encourage patients to seek help when needed.

### User Testing Results

User testing (completed with separate participants than from the focus group) was designed to identify problems with the mPCST protocol and videoconferencing technology. Overall, participants reported enjoying the relationship they fostered with their therapist first in-person and then by videoconferencing. Participants noted that they looked forward to sessions and appreciated having someone to talk to about their pain and progress each week. Progressive muscle relaxation and goal-setting skills were reported to be especially helpful. Although participants acknowledged that some skills presented were new, they described that it was helpful to receive a refresher course for previously learned skills (eg, problem solving, goal setting) to remind them of the importance of the skill and how the skill applied to pain management.

Overall, positive feedback was common, yet 2 noteworthy criticisms were reported. One participant believed the 6-session protocol felt too condensed for the amount of information delivered. Another participant recommended we tailor the sessions to suit the individual needs of different patients, for example, spending more or less time on certain sessions depending on the patient’s specific needs and preferences. Three testers reported difficulties in using the iPad and connecting with their therapist via videoconferencing; these difficulties were resolved and all participants were able to finish the protocol. We added visual information to the directions for the protocol based on these technical difficulties. All participants reported videoconferencing to be more convenient than in-person pain coping skills training sessions. Two of the 7 user-testers indicated it would be beneficial to also include the caregiver in these sessions due to the stress experienced by caregivers of HCT patients. Along these lines, 1 patient indicated the title of the protocol could be changed to “Pain and Stress Coping Skills Training” due to the relevance of the coping skills introduced in the intervention for both pain and stress management. Table 1 provides an overview of the mPCST content and how the mPCST content was adapted for HCT patients.

### Pilot Randomized Controlled Trial Results

The pilot RCT participants (n=36; different than all previous participants) were on average 56 (SD 12) years old and 50% female (18/36). Most participants were white (83%, 30/36) and 17% were black (6/36). The majority of participants were married (81%, 29/36) and just over half (56%, 20/36) had a college and/or professional degree. Most participants received an autologous HCT (83%, 30/36); 61% (22/36) had been diagnosed with multiple myeloma, 19% with lymphoma (7/36), and the remaining with various other hematological diseases. The average time since cancer diagnosis was 22 months (SD 30). Participants reported 1 other medical comorbidity, on average, with hypertension (28%, 10/36), osteoarthritis (14%, 5/36), diabetes (11%, 4/36), and sciatica (11%, 4/36) being the most common. There were no significant differences in medical or sociodemographic variables by the treatment group.

**Table 1:** Mobile pain coping skills training (mPCST) protocol adaptations made for hematopoietic stem cell transplantation (HCT) patients.

Session	Pain coping skills content	Adaptations for mPCST^a^ for HCT^b^ patients
1	Psychoeducation on pain Gate-control theory Progressive muscle relaxation	HCT patients pain experiences Audio of relaxation Relaxation video didactic Track daily pain
2	Mini-relaxation practice Imagery for relaxation	Procedure-related mini-relaxation Mini-practices in routine returning home Pair mini-practices with lifestyle recommendations (eg, walking) to increase use of both
3	Activity rest cycle Pleasant and meaningful activity planning	Activities that HCT patients report overdoing Conceptual addition of meaningful activities Activities suggested by HCT patients Volunteer activity ideas Physical activity for HCT patients
4	Examining unhelpful thoughts	Pain related thoughts reported by other HCT patients
5	Problem solving	Theme of life after transplant Pain related challenges for HCT patients Pain management suggestions from other HCT patients Training in asking for support from family and friends General advice from other HCT patients
6	Moving forward	Life priorities New life goals reported by HCT patients Training in shifting goals in response to physical health

^a^mPCST: mobile pain coping skills training.

^b^HCT: hematopoietic stem cell transplantation.

**Figure 5:**
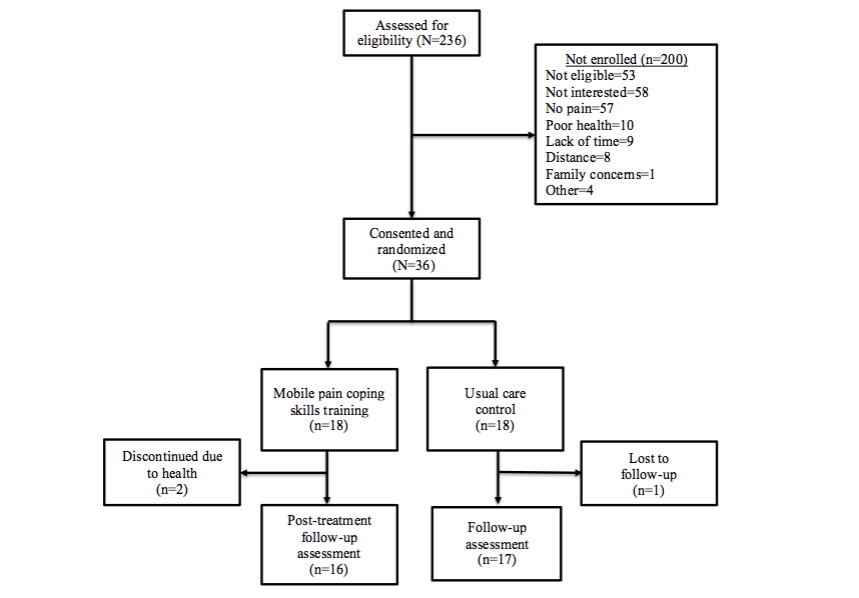
Pilot randomized controlled trial consort diagram.

The consort diagram for the RCT is presented in Figure 5. Ninety percent (36/40) of the intended participants were recruited during the proposed study timeframe. Of the 36 participants recruited, 92% completed the study (n=33). Of the 3 non completers, 2 were randomized to the intervention group and 1 was randomized to the control group. Reasons for noncompletion included patient illness and loss to follow-up. The Mann-Whitney *U*-test or Fisher exact test, whichever was appropriate, was used to determine if the baseline characteristics of noncompleters systematically differed from participants completing the study. There were no significant differences in the baseline sociodemographic characteristics between individuals who completed the study and those who did not. However, noncompleters reported significantly greater pain severity at baseline when compared with completers (mean 6.17 vs 3.14; Mann-Whitney *U*=13.50, *P*=.03).

Out of all the patients accrued, 50% (18/36) were randomized to the intervention group. Patients in the intervention group completed an average of 5 of the 6 sessions offered to them. A total of 14 participants completed all 6 sessions (1 in-person and 5 via videoconferencing); on average, these participants completed the intervention in 34 days (SD 5). Following the final session, 85% of participants reported using the skills they had learned on several days of the week. Participants reported the sessions to be helpful (mean 8/10), easy to understand (mean 7/7), and highly acceptable (mean 4/4). Overall, 75% participants rated the intervention as excellent and 25% rated it as good.

At baseline, there were no significant differences in outcome variables (ie, pain severity, pain disability, pain self-efficacy, fatigue, and physical disability [ie, self-report, 2MWT]) between randomization groups. Within-group comparisons from baseline to postintervention are presented in Table 2. Individuals in the intervention group saw improvements in all variables of interest. The pattern of effect sizes suggests that individuals in the intervention group showed greater improvements in pain disability (*d*=0.79 vs 0.69), pain self-efficacy (*d*=0.61 vs 0.10), fatigue (*d*=0.94 vs 0.81), and on the 2MWT (*d*=0.66 vs 0.41), an objective assessment of physical disability. Although differences between the outcomes are fairly subtle, the largest relative difference between the intervention and control groups appears to be for pain self-efficacy, which is a natural intermediate outcome that the intervention was designed to address directly. The magnitude of the effect sizes was greater for the control group with regard to self-reported physical disability and pain severity; however, both groups evidenced large and small-to-medium effect sizes, respectively, on these variables.

**Table 2:** Comparative pre- and postintervention data.

Outcome variables		Pretreatment, mean (SD)	Post-treatment, mean (SD)	Mean difference (SD)	95% CI	*P* value	*d*
**Intervention (n=16)**							
	Pain severity	3.00 (2.09)	2.69 (1.89)	0.31 (1.20)	−0.33 to 0.95	.32	0.26	
	Pain disability	30.26 (13.97)	19.22 (12.59)	11.04 (13.92)	3.48 to 3.63	.006^a^	0.79
	Pain self-efficacy	60.50 (23.39)	77.13 (19.39)	−16.63 (27.42)	−31.24 to −2.01	.03^b^	0.61
	Fatigue	18.60 (5.75)	13.27 (4.89)	5.33 (5.68)	2.19 to 8.48	.003^a^	0.94	
	Physical disability	12.36 (4.11)	16.11 (4.14)	−3.76 (4.56)	−6.19 to −1.32	.005^b^	0.82
	2-min walk test	125.88 (19.69)	134.94 (19.05)	−9.06 (13.75)	−17.00 to −1.12	.03^a^	0.66
**Treatment as usual (n=17)**							
	Pain severity	3.28 (2.40)	2.50 (1.87)	0.78 (1.67)	−0.08 to 1.64	.07	0.47
	Pain disability	23.84 (20.11)	18.00 (15.84)	5.84 (8.50)	1.31 to 10.38	.02^b^	0.69
	Pain self-efficacy	61.53 (25.52)	63.76 (24.52)	−2.24 (21.72)	−13.40 to 8.93	.68	0.10
	Fatigue	19.29 (4.83)	15.35 (5.41)	3.94 (4.88)	1.43 to 6.45	.004^a^	0.81	
	Physical disability	11.72 (5.76)	16.61 (3.85)	−4.89 (3.80)	−6.84 to −2.93	<.001^a^	1.29
	2-min walk test	121.12 (19.24)	126.99 (21.71)	−5.87 (14.19)	−15.40 to 3.67	.20	0.41

^a^*P*<.01.

^b^*P*<.05.

## Discussion

### Primary Outcomes

The goal of this study was to develop a PCST intervention that could be used to enhance the ability of HCT patients to manage their pain following transplant. HCT patients face many pain-related challenges and challenges accessing behavioral interventions post-transplant. We used both patient and provider focus groups to adapt the effective components of existing PCST protocols to meet the unique needs of patients following transplant. After developing the HCT-focused protocol, we conducted a small RCT to examine feasibility, acceptability, and initial efficacy of the developed mPCST protocol.

The developed mPCST protocol included 1 in-person session at the medical center between the patient and study therapist, which led to the development of a successful working relationship and the integration of mPCST with the patient’s medical care. Then, once the patient returned home following intensive outpatient care, 5 more sessions were conducted using videoconferencing. This bridge between hospitalization and home maintained the continuity of care as patients moved away from the medical center. This mixed delivery modality fostered a strong patient-therapist relationship that likely increased the feasibility, acceptability, and efficacy of the intervention. To our knowledge, this is one of the first studies that has examined the use of videoconferencing to provide patients with PCST upon their return home from inpatient or intensive outpatient care.

Patient focus groups highlighted several areas of consideration in the development of the intervention content including the importance of strategies for coping with pain that was unique to HCT patients, enjoyable activities following transplant, pain-related thoughts following HCT, and connecting with other HCT patients for support. Accordingly, we addressed many of these areas in the developed mPCST protocol. For example, we highlighted advice from other patients throughout the sessions and in patient materials. Although we were unable to include all patient suggestions (eg, incorporate a caregiver as an active member of the intervention, tailoring the protocol to meet the specific needs of each patient) into the protocol, we will consider these suggestions in our future work.

Provider focus group information was used as one of the last steps in refining the intervention protocol and website. Providers emphasized the importance of helping participants track and manage not only their pain but also their fatigue. They also suggested that participants wear pedometers to track daily steps. We incorporated these suggestions into this study by modifying the study website to collect daily pain, fatigue, and steps; we used the data clinically to help patients increase their activity by providing feedback in the *Just for You* portion of the website (see Figure 4). In our planned future work, we will incorporate daily reports of pain, fatigue, and steps to tailor the intervention content to meet patients’ specific needs. As technology use has advanced from primarily Web-based platforms to mobile phone-based platforms, it will be important to consider data entry (eg, through apps) on hand-held devices and the provision of immediate feedback through programming (eg, push notifications) in future work. In the time since provider focus groups and the end of this study, there has been evidence that providers find the mPCST protocol to be feasible and acceptable to patients. First, HCT providers have continued to refer HCT patients for pain coping skills to our clinical practice. Second, the larger transplant program is working with our pain program to incorporate videoconferencing pain coping skills and other psychosocial services for HCT patients in both their clinical practice and their research protocols.

The pilot RCT was designed to put us in a good position to perform a more definitive RCT in a subsequent follow-up study. Apart from assessing the feasibility of the study methods, we asked the following 2 questions: (1) would the intervention group improve and, if so, how much, and also which outcomes would show the greatest improvement, and (2) how much improvement would we see in the control group? This latter question is critical for the design of the more definitive RCT. We used a small pilot RCT to examine the pattern of effects for those who received mPCST and those who did not. We found that participants who received the intervention experienced greater improvements in pain disability, pain self-efficacy, fatigue, and the 2MWT, with the largest relative difference between the intervention and control groups being for pain self-efficacy and the 2MWT. The finding that HCT patients completed the mPCST intervention at the intended pace of about 1 session per week adds support for past work suggesting that videoconferencing intervention protocols compared with in-person protocols can be completed at the pace of 1 session per week, whereas in-person protocols can take twice as long [56].

These outcomes suggest that mPCST is particularly likely to help patients manage their pain in a way that might lead to decreases in the impact of pain on their day-to-day activities (ie, pain disability). In line with social cognitive theory, teaching and receiving feedback on pain coping skills practice in their own home (vs a medical center setting) may have been particularly helpful for increasing participants’ self-efficacy for pain management. Furthermore, the finding that an objective measure of the physical ability (ie, the two-min walk test) appears to have been positively impacted by mPCST has important implications for improving patients’ physical functioning in their daily activities. It may also be said that helping patients control their pain and other symptoms can lead to increased physical activity.

Both the groups evidenced small-moderate effect sizes for pain severity. The majority of individuals participating in the RCT had been diagnosed with multiple myeloma (62%) for which autologous transplantation is most commonly recommended [56]. Complete remission is achieved in only about half of all cases following autologous transplantation [56]. Persistent pain (ie, in bone) is common among these patients, and may result from the disease, therapeutic interventions, or indicate disease progression [56-60]. Typically, pain is associated with functional limitations [57] as well as increased mood disturbance [61]. Although large changes in ratings of pain severity were not found, participants receiving the intervention experienced greater improvements in self-efficacy for pain control and pain disability when compared with participants in the control group. Given the chronic nature of multiple myeloma and persistence of pain in this population, an intervention that improves the patients’ confidence in their ability to manage their pain and decrease pain disability early in their disease trajectory may be critical for helping them better manage long-term complications.

The small effect sizes for pain severity ratings may indicate that it is necessary to target patients with higher baseline pain levels to find a change in pain severity. The average pain severity of patients recruited in this study was 3 on a 10-point scale; this relatively low pain level may not have been high enough to demonstrate significant change from pre- to postintervention. Another important consideration is that patients with higher pain were less likely to complete the study and may have been less likely to enroll; future work may want to consider recruitment and retention strategies to provide treatment to individuals with the highest pain levels who may need it the most. Finally, there is evidence that changes in self-efficacy for pain control and pain disability are critical for overall functioning and as such, investigators may want to consider using these variables as primary outcome variables with actual pain severity being a secondary outcome.

### Limitations

This study has several limitations. First, this work was completed through the use of iPads, which require either a data plan or Internet connection to be able to videoconference with the therapist. The intervention itself is scalable for use on either a personal computer or mobile phone; the number of individual’s access to a personal computer, tablet, or mobile phone is greater than 50%, and future work should be designed to be inclusive of all possible technology devices. Second, the RCT was relatively small (N=36), conducted in a single medical center, and had a short follow-up period; future work should expand the study size, consider using multiple sites, and examine longer term outcomes.

### Conclusions

In summary, this study relied on past work and the expertise of the large study team to develop a mobile pain coping skills training intervention that was efficacious and could be delivered to patients following HCT. The developed intervention was also informed by patient and provider focus groups and included a 6-session, hybrid protocol (ie, in-person and videoconferencing). Our pilot RCT found that the developed intervention was highly feasible and acceptable to HCT patients with pain. Preliminary data suggest that the developed mPCST intervention likely improves patients’ abilities to manage their pain (ie, pain self-efficacy), decrease pain-related disability, and decrease symptoms of fatigue.

## Abbreviations

ABMT: adult bone marrow transplant clinic
CSQ: Client Satisfaction Questionnaire
FACT: Functional Assessment of Cancer Therapy well-being
HCT: hematopoietic stem cell transplantation
mHealth: mobile health
mPCST: mobile pain coping skills training
PCST: pain coping skills training
RCT: randomized controlled trial
2MWT: 2-min walk test

## Appendix

Multimedia Appendix 1 CONSORT-EHEALTH checklist (V 1.6.1).
